# Effects of Sources and Forms of Vitamin K_3_ on Its Storage Stability in Vitamin Premixes or Vitamin Trace Mineral Premixes

**DOI:** 10.3390/ani11041140

**Published:** 2021-04-16

**Authors:** Huakai Wang, Pan Yang, Longxian Li, Nan Zhang, Yongxi Ma, Xuexin Xu

**Affiliations:** State Key Laboratory of Animal Nutrition, College of Animal Science and Technology, China Agricultural University, Beijing 100193, China; huakaiwhk@cau.edu.cn (H.W.); ypan23@163.com (P.Y.); S20193040586@cau.edu.cn (L.L.); S20203040629@cau.edu.cn (N.Z.); 13601089425@163.com (X.X.)

**Keywords:** vitamin K_3_, stability, storage time, temperature-relative humidity, chloride choline, premix

## Abstract

**Simple Summary:**

Vitamin K_3_ (VK_3_) is the most unstable vitamin. Menadione sodium bisulfite (MSB) and menadione nicotinamide bisulfite (MNB) are two commonly used VK_3_ in animal diets. Micro-capsule and micro-sphere forms of vitamins, provided from encapsulation techniques, have been used in vitamin production, while the effects of these encapsulated forms on the storage stability of VK_3_ need systematic investigation. Our results show that high temperature-high relative humidity had a negative effect on the recovery of VK_3_. The retention of MNB was higher than that of MSB in vitamin premixes. VK_3_ retention was higher in micro-capsule or micro-sphere forms in vitamin premix and vitamin trace mineral premixes during storage.

**Abstract:**

Six types of vitamin K_3_ (VK_3_); two sources (menadione sodium bisulfite, MSB; menadione nicotinamide bisulfite, MNB), and three different forms (crystal, micro-capsule, and micro-sphere) were used to determine the retention of VK_3_ in vitamin premixes (Experiment 1) or vitamin trace mineral (VTM) premixes (Experiment 2) after 1, 2, 3, and 6 months of storage. The retention of VK_3_ in vitamin premixes was evaluated at 25 °C/60% relative humidity or 40 °C/75% relative humidity in an incubator in Experiment 1 and in VTM premixes (choline chloride: 0 vs. 16,000 mg/kg) stored at room temperature in Experiment 2. The VK_3_ retention in vitamin premix or VTM premix decreased significantly with the extension of storage time (*p* < 0.05). In Experiment 1, the VK_3_ retention was higher in the 25 °C/60% incubator (56%) than in the 40 °C/75% incubator (28%). The MNB retention (52%) was higher than MSB retention (32%). The retention of VK_3_ in micro-capsules (43%) or micro-spheres (48%) was higher than the crystal form (35%) after six months of storage. In Experiment 2, there was no difference between the retention of MSB (49%) or MNB (47%). The retention of VK_3_ of micro-capsule (51%) or micro-sphere (54%) was higher than that of crystal form (40%). The VK_3_ retention was higher in the choline-free group (51%) than in the choline group (47%) after six months of storage. Finally, the predicted equations of VK_3_ retention with storage time in vitamin premixes or VTM premixes were established. The *R*^2^ of the prediction equations was ≥0.9005, indicating that time is an important factor in predicting VK_3_ retention. In conclusion, the higher temperature-relative humidity, choline had negative effects on VK_3_ retention during premix storage. MNB retention was higher than MSB during storage of vitamin premix. The encapsulated forms of VK_3_, micro-capsules and micro-spheres, could improve VK_3_ storage stability in vitamin premix and VTM premix.

## 1. Introduction

Vitamins are involved in the metabolism of animals and have positive effects on animal health [1]. However, proper integration is difficult to achieve due to the instability of many vitamins with physical and chemical factors [2]. The presence of choline and improper storage environment cause losses of vitamins in premixes. In previous studies on vitamin stability, we found that VK_3_ was the most unstable vitamin [3], which could directly affect the effects of vitamin premix and can be used as a marker of the validity of the premix. The encapsulated forms of VK_3_, such as micro-capsule or micro-sphere form was chosen by some premix manufacturers to improve VK_3_ stability, but more research is needed to validate their storage stability [4]. Considering the difference in climate between North China and South China, we also used temperature-relative humidity as a factor when designing the experiment. Therefore, the aim of this study was to determine the influence of two sources and three forms of VK_3_, temperature- relative humidity and choline chloride, on the storage stability of VK_3_ in vitamin and vitamin trace mineral (VTM) premix, and develop predicted equations to estimate VK_3_ retention during storage, for accurate usage of VK_3_ in the feed industry and animal production.

## 2. Materials and Methods

This study was conducted at the Ministry of Agriculture and Rural Affairs Feed Efficacy and Safety Evaluation Center located at China Agricultural University (Beijing, China).

### 2.1. Premix Treatments

Six types of VK_3_ (Provided by Wellroad Animal Health Co. Ltd., Taiyuan, China) were used, and the analyzed VK_3_ content in these forms is shown in Table S1. Experiment 1 was designed by a 2 × 3 × 2 factorial arrangement with two sources, menadione sodium bisulfite (MSB) vs. menadione nicotinamide bisulfite (MNB), and three forms (crystal vs. micro-capsule vs. micro-sphere), and two storage environments (temperature/relative humidity: 25 °C/60% vs. 40 °C/75%). Six vitamin premixes were formulated using different VK_3_ (Table 1). Experiment 2 was designed with a 2 × 3 × 2 factorial arrangement with two sources (MSB vs. MNB), three forms (crystal vs. micro-capsule vs. micro-sphere), and with and without choline (0 vs. 16,000 mg/kg). Twelve vitamin trace mineral (VTM) premixes were formulated (with and without choline), which contained the same levels of vitamins except for choline (Table 1). Vitamin levels were determined based on typical commercial premixes [5,6] to make the laboratory analysis more reliable.

### 2.2. Premix Preparation, Storage, and Sampling

Premixes were formulated at Wellroad Animal Health Co. Ltd., Taiyuan, China. Each of the vitamin premixes was prepared in six separate batches following the same procedure (6.0 kg per batch). Each batch represented one repetition and was divided into ten polyethylene bags weighing 0.6 kg. Each of the twelve VTM premixes was prepared in six separate batches following the same procedure (15.0 kg per batch). Each batch represented one repetition and was divided into five polyethylene bags (3.0 kg). The vitamin premixes were stored in two incubators (Cerberus instrument Co., Ltd., Beijing, China), with the temperature/relative humidity set at 25 °C/60% or 40 °C/75%. Thirty bags of vitamin premixes were randomly placed in each incubator. The VTM premixes were stored in a storeroom, the temperature and relative humidity of the storeroom were recorded daily (Figure S1). The samples were analyzed after 0, 1, 2, 3, and 6 months of storage.

### 2.3. Determination of Vitamin K_3_ Content

The content of VK_3_ in premix was determined with high-performance liquid chromatography (HPLC) [7]. In brief, a 0.5 g sample was extracted with 50 mL of trichloromethane. Then, 5 mL sodium carbonate solution and 5 g adsorbent were added, and after 30 min of blending, the extract passed 0.45 μM filter samples were assayed in triplicates. The VK_3_ retention was used as the stability measurement index, based on a method in a previous study [3] as follows: the retention of VK_3_ (%) = (nutrient content per gram of premix after storage × premix weight (gram) after storage)/(nutrient content per gram of premix × premix weight (gram) before storage) × 100.

### 2.4. Statistical Analysis

Statistical analysis was performed using a Proc MIXED model of SAS (SAS Institute, Cary, NC, USA). The statistical model included the fixed main effects of source, form, temperature/relative humidity, and their interaction effects in Experiment 1. The statistical model included the fixed main effects of source, form, choline, and their interaction effects in Experiment 2. A single degree of freedom contrast was performed for fixed effects in Experiment 1 and 2. Meanwhile, statistical differences among mean values were separated by Tukey’s multiple comparison test (Tables S2 and S3). Statistical significance was considered at *p* < 0.05. Excel 2019 (Microsoft Corporation, Redmond, Washington, DC, USA) was used to establish the prediction equation. The *R*^2^ and root mean square error (RMSE) were used to examine the quality of the prediction equation.

## 3. Results

The analyzed VK_3_ values of the samples are shown in Table 2, which was consistent with that of the research design.

### 3.1. Effect of Source and Form of Vitamin K_3_ and Temperature/Relative Humidity on Its Stability in Vitamin Premix during Storage

The retention of VK_3_ was significantly decreased with the extension of the storage time in vitamin premix (*p* < 0.05, Table 3). The retention of MNB (86%, 80%, 73%, or 52% after 1, 2, 3, or 6-months of storage, respectively) was significantly higher than that of MSB (82%, 66%, 54%, or 32% after 1, 2, 3, or 6-months of storage, respectively) during storage (*p* < 0.05). The VK_3_ retention of micro-capsule or micro-sphere was significantly higher than crystals (*p* < 0.05) at each time point. The VK_3_ retention was significantly higher at 25 °C/60% relative humidity than 40 °C/75% relative humidity (*p* < 0.05). In addition, there were interaction effects between source, form, and temperature-relative humidity after part of the storage time (*p* < 0.05). Although there was no significant difference between the retention of MSB of micro-capsules (71%) and micro-spheres (73%), the retention of MNB of micro-capsules (74%) was lower than the MNB of micro-spheres (80%) at 40 °C/75% relative humidity after one month of storage (*p* < 0.05, Table S2), this suggested that there was an interaction between the source and form. In addition, there was no difference between the retention of MSB micro-capsules (88%) and that of MNB (89%) at 25 °C/60% relative humidity, while the retention of MNB micro-capsules (65%) was higher than that of MSB (50%) at 40 °C/75% after two months of storage (*p* < 0.05, Table S2), this demonstrated that there was an interaction between the source and temperature.

### 3.2. Effect of Source and Form of Vitamin K_3_ and Choline on Its Stability in Vitamin Trace Mineral Premix during Storage

The retention of VK_3_ was significantly decreased with the extension of storage time in VTM premix (*p* < 0.05, Table 4). There was no statistical difference between the retention of MSB (95%, 88%, 83%, or 49%) and MNB (94%, 87%, 81%, or 47%) after one, two, three, or six months of storage. The retention of VK_3_ of micro-capsule or micro-sphere was significantly higher than crystals (*p* < 0.05) at each time of the investigation. Furthermore, the retention of VK_3_ in a choline-free group (51%) was higher (*p* < 0.05) than that of the choline group (46%) after six months of storage. In addition, there were interaction effects between source, form, and choline after part of the storage time (*p* < 0.05). For example, there was no difference between the retention of MNB crystals in the choline-free group (71%) and the choline group (73%), while the retention of MSB crystals in the choline-free group (84%) was higher than that of MSB in choline group (75%) after three months of storage (*p* < 0.05, Table S3), this indicated that there was an interaction between the source and choline. Meanwhile, the retention of MSB of micro-sphere in the choline-free group (84%) was lower than that of MSB in the choline group (90%), while the retention of MSB of crystal in the choline-free group (84%) was higher than that of MSB in choline group (75%) after three months of storage (*p* < 0.05, Table S3), this suggested that there was an interaction between the form and choline.

### 3.3. Prediction Equations for Retention of Vitamin K_3_ in Vitamin or Vitamin Trace Mineral (VTM) Premixes during Storage

**Figure 1:**
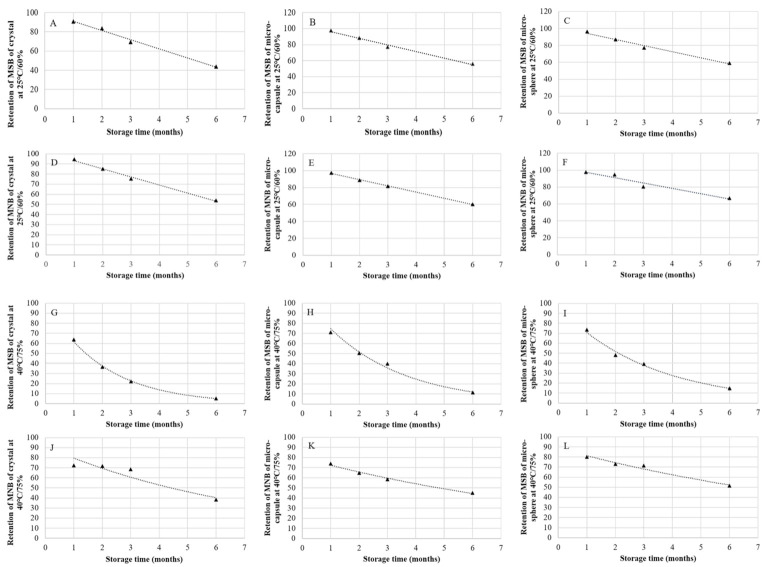
Prediction equations for vitamin K_3_ retention (%) in vitamin premixes during storage. (**A**) Retention of MSB of crystal at 25 °C/60% (y = −9.5807x + 100.58, *R*^2^ = 0.992); (**B**) Retention of MSB of micro-capsule at 25 °C/60% (y = −8.255x + 104.43, *R*^2^ = 0.990); (**C**) Retention of MSB of micro-sphere at 25 °C/60% (y = −7.2871x + 101.66, *R*^2^ = 0.985); (**D**) Retention of MNB of crystal at 25 °C/60% (y = −8.0286x + 101.26, *R*^2^ = 0.994); (**E**) Retention of MNB of micro-capsule at 25 °C/60% (y = −7.3439x + 103.92, *R*^2^ = 0.999); (**F**) Retention of MNB of micro-sphere at 25 °C/60% (y = −6.3364x + 103.8, *R*^2^ = 0.943); (**G**) Retention of MSB of crystal at 40 °C/75% (y = 101.26e − 0.498x, *R*^2^ = 0.999); (**H**) Retention of MSB of micro-capsule at 40 °C/75% (y = 107.69e − 0.367x, *R*^2^ = 0.991); (**I**) Retention of MSB of micro-sphere at 40 °C/75% (y = 96.72e − 0.313x, *R*^2^ = 0.994); (**J**) Retention of MNB of crystal at 40 °C/75% (y = 91.176e − 0.136x, *R*^2^ = 0.901); (**K**) Retention of MNB of micro-capsule at 40 °C/75% (y = 79.747e − 0.097x, *R*^2^ = 0.991); (**L**) Retention of MNB of micro-sphere at 40 °C/75% (y = 88.783e − 0.088x, *R*^2^ = 0.974). MSB, menadione sodium bisulfite; MNB, menadione nicotinamide bisulfite.

**Figure 2:**
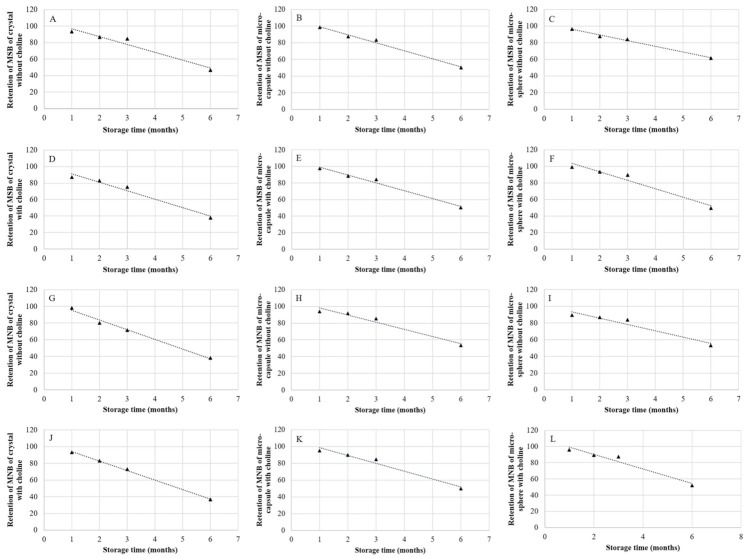
Prediction equations for vitamin K_3_ retention (%) in vitamin trace mineral (VTM) premixes during storage. (**A**) Retention of MSB of crystal without choline (y = −9.5393x + 106.29, *R*^2^ = 0.951); (**B**) Retention of MSB of micro-capsule without choline (y = −9.5707x + 108.66, *R*^2^ = 0.988); (**C**) Retention of MSB of micro-sphere without choline (y = −6.8729x + 103.11, *R*^2^ = 0.991); (**D**) Retention of MSB of crystal with choline (y = −10.227x + 101.33, *R*^2^ = 0.970); (**E**) Retention of MSB of micro-capsule with choline (y = −9.4579x + 108.52, *R*^2^ = 0.983); (**F**) Retention of MSB of micro-sphere with choline (y = −10.225x + 113.7, *R*^2^ = 0.954); (**G**) Retention of MNB of crystal at without choline (y = −11.606x + 106.85, *R*^2^ = 0.989); (**H**) Retention of MNB of micro-capsule without choline (y = −8.5214x + 106.62, *R*^2^ = 0.958); (**I**) Retention of MNB of micro-sphere without choline (y = −7.5507x + 100.93, *R*^2^ = 0.938); (**J**) Retention of MNB of crystal with choline (y = −11.378x + 105.59, *R*^2^ = 0.998); (**K**) Retention of MNB of micro-capsule with choline (y = −9.3086x + 107.79, *R*^2^ = 0.968); (**L**) Retention of MNB of micro-sphere with choline (y = −8.9407x + 108.06, *R*^2^ = 0.952). MSB, menadione sodium bisulfite; MNB, menadione nicotinamide bisulfite.

Prediction equations have been used in many aspects of animal nutrition, such as the prediction of digestibility of feed ingredients and enzyme activity [8,9]. The use of prediction equations can decrease experimental costs and improve the accuracy when estimating values. The prediction equation with the greatest *R*^2^ and the smallest *RMSE* was regarded as the most accurate prediction equation [8]. The prediction equations in this research were fitted with data, and all of the equations had a good fit (Figure 1 and Figure 2). Time is an important predictor of vitamin recovery during storage, which is consistent with one former research [3].

## 4. Discussion

### 4.1. Effect of Storage Time on the Stability of Vitamin K_3_

Storage time is an important factor affecting the retention of VK_3_ in vitamin premixes and VTM premixes. European Food Safety Authority [10] reported that after one month of storage, VK_3_ retained 88.65% or 83.38% of its original activity in vitamin premix or VTM premix, respectively. After three months, VK_3_ retained 77.00% or 67.80% of its original activity in vitamin premix or VTM premix, respectively. Shurson et al. [11] observed that the retention of VK_3_ was 75.96% in vitamin premix after 120 d storage. Whitehead [12] reported that the retention of VK_3_ was 64% or 0% after one or six months in VTM premix. All these reports had a similar outcome to our research. The outcome was mainly due to the degradation of VK_3_ and a reduction in its effective active ingredient with the extension of the storage time. Therefore, we recommend reducing the time interval between the production and use of vitamin premix or VTM premix.

### 4.2. Effect of Source on the Stability of Vitamin K_3_

MSB is a free-flowing white to yellow crystalline, almost odorless powder that is extremely unstable when exposed to air and sensitive to light and heat. Due to its instability, a new K_3_ preparation, MNB, was developed. The MNB was developed by combining nicotinamide molecular groups to replace the sodium ion and three crystalline water molecules in MSB. European Food Safety Authority [10] reported that MSB or MNB had a shelf life of 18 or 30 months at 25 °C. In addition, they indicated that the MNB retention was 93.1% or 82.3%, and the MSB retention was 84.2% or 71.1% after one month or three months of storage, respectively. Our results also showed that MNB retention was higher than MSB after storage in vitamin premix, which was consistent with previous research. The difference between MSB retention and MNB retention may be explained by their chemical structure. MSB has three more crystalline water molecules than MNB, and the presence of water molecules more easily triggers the degradation of VK_3_ [13]. However, there was no difference between the retention of MSB and MNB during storage in VTM premix. This may be related to the ambient temperature and relative humidity, composition of premixes, and the microencapsulation process in the present study.

### 4.3. Effect of Form on the Stability of Vitamin K_3_

Encapsulation preparation techniques have been used in vitamin production in recent years, and the benefit of these technologies can extend the shelf-time of vitamins, control release in the intestine, and increase bio-availability [14,15]. As the capsule core of the capsule, vitamins reduce the contact with unfavorable factors such as air and moisture, so its stability is improved. Coelho [2] reported that the retention of crystalline MSB was 84%, 75%, 66%, 40%, and the retention of coated MSB was 92%, 86%, 80%, 68%, in vitamin premixes after one, two, three or six months of storage, respectively. In a study by Coelho [2], the stability of coated MSB was higher than crystalline MSB. These results were consistent with our observation, which showed that micro-capsule and micro-sphere forms of VK_3_ provided by encapsulation and micro-particle preparation techniques might improve its stability during storage.

### 4.4. Effect of Temperature and Relative Humidity on the Stability of Vitamin K_3_

The temperature and relative humidity are important factors affecting the stability of VK_3_ in premix. Our results showed that the retention of VK_3_ was higher at 25 °C/60% than at 40 °C/75%. This was mainly due to high temperature provides energy for many redox reactions and accelerates the destruction of VK_3_ [16]. In addition, the increase in relative environmental humidity increased the absorption of water by VK_3_, creating conditions for the destruction of oxygen and trace elements, causing VK_3_ degradation [13]. Therefore, vitamin premix should be stored under lower temperature and relative humidity conditions.

### 4.5. Effect of Choline on the Stability of Vitamin K_3_

Choline has strong hygroscopicity and can absorb moisture from the environment, which can affect the stability of the other vitamin [17]. Our results showed that the VK_3_ retention was higher in the choline-free group than in the choline group after six months of storage in VTM premix. Tavčar-Kalcher et al. [18] reported that the retention of VK_3_ was 80% in a choline-free group and 9% in a choline group after twelve months of storage of the vitamin premix. In an experiment by Coelho [2], the retention of VK_3_ was 94% in a choline-free group and 40% in a choline group in vitamin premix after six months of storage. These results were consistent with the present study, indicating that choline significantly affected the stability of VK_3_ during long-term storage. This is mainly due to the fact that choline absorbs a large amount of water from the environment, putting VK_3_ in an imbalanced state which accelerates its degradation. Therefore, vitamin stability could be improved by removing choline chloride from premixes.

## 5. Conclusions

Our results showed that MNB was a better source of VK_3_ in vitamin premix, while VK_3_ performed better when coated in vitamin premix and VTM premix. Meanwhile, the VTM premix should not contain choline during storage, and the vitamin premix and VTM premix should be stored at low temperature and relative humidity conditions to ensure the stability of VK_3_. Additionally, the prediction equations obtained in this study may be adopted by vitamin and/or VTM premix companies and their users for predicting the retention of VK_3_ for a more accurate nutrient allowance in animal production.

## Figures and Tables

**Table 1:** Composition of the vitamin and vitamin-trace mineral (VTM) premixes.

Item	Vitamin Premix	VTM (Choline-Free) Premix	VTM (Choline) Premix
Vitamin, unit/kg			
Vitamin A, IU	12,000,000	2,400,000	2,400,000
Vitamin D_3_, IU	4,000,000	800,000	800,000
Vitamin E, IU	100,000	20,000	20,000
Vitamin K_3_^1^, mg	5000	1000	1000
Vitamin B_1_, mg	4000	800	800
Vitamin B_2_, mg	10,000	2000	2000
Vitamin B_6_, mg	6000	1200	1200
Vitamin B_12_, mg	50	10	10
Niacin, mg	55,000	11,000	11,000
Pantothenic acid, mg	30,000	6000	6000
Biotin, mg	250	200	200
Folic acid, mg	1000	50	50
Choline chloride, mg		−	16,000
Trace Mineral, mg/kg			
Cu (CuSO_4_)		4000	4000
Fe (FeSO_4_)		20,000	20,000
Zn (ZnO)		20,000	20,000
Mn (MnO)		6000	6000
I (Ca (IO_3_)_2_)		200	200
Se (NaSeO_2_)		60	60

^1^ Six types of VK_3_ were MSB of crystal, MSB of micro-capsule, MSB of mic-sphere, MNB of crystal, MNB of micro-capsule, MNB of micro-sphere. MSB, menadione sodium bisulfite; MNB, menadione nicotinamide bisulfite.

**Table 2:** Analyzed vitamin K_3_ values in vitamin and vitamin vitamin-trace mineral (VTM) premixes.

Types	Source	Form	T-H	Choline	Effective Content (mg/kg) ^1^
Vitamin premix 1	MSB	crystal	25 °C/60%	−	4990
Vitamin premix 2	MSB	micro-capsule	25 °C/60%	−	5001
Vitamin premix 3	MSB	micro-sphere	25 °C/60%	−	4910
Vitamin premix 4	MNB	crystal	25 °C/60%	−	5043
Vitamin premix 5	MNB	micro-capsule	25 °C/60%	−	5011
Vitamin premix 6	MNB	micro-sphere	25 °C/60%	−	5031
Vitamin premix 7	MSB	crystal	40 °C/75%	−	5019
Vitamin premix 8	MSB	micro-capsule	40 °C/75%	−	4991
Vitamin premix 9	MSB	micro-sphere	40 °C/75%	−	5018
Vitamin premix 10	MNB	crystal	40 °C/75%	−	5020
Vitamin premix 11	MNB	micro-capsule	40 °C/75%	−	5033
Vitamin premix 12	MNB	micro-sphere	40 °C/75%	−	5010
VTM premix 1	MSB	crystal	−	−	958
VTM premix 2	MSB	crystal	−	+	1042
VTM premix 3	MSB	micro-capsule	−	−	973
VTM premix 4	MSB	micro-capsule	−	+	993
VTM premix 5	MSB	micro-sphere	−	−	990
VTM premix 6	MSB	micro-sphere	−	+	1073
VTM premix 7	MNB	crystal	−	−	988
VTM premix 8	MNB	crystal	−	+	1059
VTM premix 9	MNB	micro-capsule	−	−	1021
VTM premix 10	MNB	micro-capsule	−	+	1057
VTM premix 11	MNB	micro-sphere	−	−	1054
VTM premix 12	MNB	micro-sphere	−	+	1001

^1^ Values represent the mean of six replicate samples, and each sample was analyzed in duplicate. MSB, menadione sodium bisulfite; MNB, menadione nicotinamide bisulfite; T-H, temperature and relative humidity; VTM, vitamin trace mineral.

**Table 3:** Effects of sources and forms of vitamin K_3_, and temperature/relative humidity on the retention of vitamin K_3_ in vitamin premixes.

VK_3_ Retention (%)/Months	Source (A)		Form (B)			T-H (C)		SEM	*p*-Value						
	MSB	MNB	Crystal	Micro-Capsule	Micro-Sphere	25 °C/60%	40 °C/75%		A	B	C	A × B	A × C	B × C	A × B × C
1	82 ^Iβ^	86 ^Iα^	80 ^Ic^	85 ^Ib^	87 ^Ia^	96 ^IA^	72 ^IB^	1.80	<0.001	<0.001	<0.001	0.011	<0.001	0.003	0.754
2	66 ^IIβ^	80 ^IIα^	69 ^IIb^	73 ^IIa^	76 ^IIa^	88 ^IIA^	57 ^IIB^	2.24	<0.001	<0.001	<0.001	<0.001	<0.001	0.958	0.002
3	54 ^IIIβ^	73 ^IIIα^	59 ^IIIb^	64 ^IIIa^	67 ^IIIa^	77 ^IIIA^	50 ^IIIB^	2.20	<0.001	<0.001	<0.001	<0.001	<0.001	0.021	<0.001
6	32 ^IVβ^	52 ^IVα^	35 ^IVc^	43 ^IVb^	48 ^IVa^	56 ^IVA^	28 ^IVB^	2.47	<0.001	<0.001	<0.001	0.224	<0.001	0.282	0.168
*p*-value	<0.001	<0.001	<0.001	<0.001	<0.001	<0.001	<0.001								

^I, II, III, IV^ Means in a column, with different superscripts indicating significance at different time points (*p* < 0.05);> ^α, β^ Means in a row, with different superscripts indicating significance differences (*p* < 0.05) of source; ^a, b, c^ Means in a row, with different superscripts indicating significant difference (*p* < 0.05) of form; ^A, B^ Means in a row, with different superscripts indicating significant differences (*p* < 0.05) between different T-H values. SEM, Standard Error of Mean; MSB, menadione sodium bisulfite; MNB, menadione nicotinamide bisulfite; A, source; B, form; C, T-H; T-H, temperature and relative humidity.

**Table 4:** Effects of sources and forms of vitamin K_3_ and choline on the retention of vitamin K_3_ in vitamin trace mineral (VTM) premixes.

VK_3_ Retention (%)/Months	Source (A)		Form (B)			Choline (C)		SEM	*p*-Value						
	MSB	MNB	Crystal	Micro-Capsule	Micro-Sphere	−	+		A	B	C	A × B	A × C	B × C	A × B × C
1	95 ^I^	94 ^I^	93 ^Ib^	96 ^Ia^	95 ^Iab^	95 ^I^	95 ^I^	0.71	0.210	0.010	0.661	<0.001	0.163	<0.001	0.787
2	88 ^II^	87 ^II^	83 ^IIb^	89 ^IIa^	89 ^IIa^	87 ^II^	88 ^II^	0.72	0.329	<0.001	0.216	0.031	0.925	0.058	0.058
3	83 ^III^	81 ^III^	76 ^IIIc^	84 ^IIIb^	86 ^IIIa^	82 ^III^	82 ^III^	0.95	0.129	<0.001	0.363	<0.001	<0.001	<0.001	<0.001
6	49 ^IV^	47 ^IV^	40 ^IVb^	51 ^IVa^	54 ^IVa^	51 ^IVA^	46 ^IVB^	1.32	0.156	<0.001	<0.001	0.064	0.105	0.449	0.026
*p*-value	<0.001	<0.001	<0.001	<0.001	<0.001	<0.001	<0.001								

^I, II, III, IV^ Means in a column, with superscripts indicating significance at different time points (*p* < 0.05); ^a, b, c^ Means in a row, with different superscripts indicating significant difference (*p* < 0.05) of form; ^A, B^ Means in a row, with different superscripts indicating significant differences (*p* < 0.05) of choline or choline-free. SEM, Standard Error of Mean; MSB, menadione sodium bisulfite; MNB, menadione nicotinamide bisulfite; A, source; B, form; C, choline.

## Data Availability

Not applicable.

## Supplementary Materials

The following are available online at https://www.mdpi.com/article/10.3390/ani11041140/s1, Table S1: The analysis of VK_3_ in six forms, Table S2: Effects of sources and forms of vitamin K_3_, and temperature/relative humidity on the retention of vitamin K_3_ in vitamin premixes, Table S3: Effects of sources and forms of vitamin K_3_ and choline on the retention of vitamin K_3_ in vitamin trace mineral (VTM) premixes, Figure S1: The record of temperature and relative humidity during the storage of the vitamin trace mineral premixes.
