# Supplementary material for: Effects of Sources and Forms of Vitamin K3 on Its Storage Stability in Vitamin Premixes or Vitamin Trace Mineral Premixes
Source: Animals (Basel). 2021 Apr 16;11(4):1140. doi: 10.3390/ani11041140 (PMC8072531; doi:10.3390/ani11041140)
Supplement: Supplementary file 1 [file animals-11-01140-s001.zip › animals-1140474-supplementary.doc]

**Table S1:** The analysis of VK_3_ in these six forms.

| **Vitamin K_3_** | **Analyzed vitamin K_3_ values (%)** |
| --- | --- |
| MSB of crystal | 50 |
| MSB of micro-capsule | 89 |
| MSB of micro-sphere | 6 |
| MNB of crystal | 44 |
| MNB of micro-capsule | 9 |
| MNB of micro-sphere | 6 |

Six types of VK_3_ were provided by the Wellroad Animal Health Co. Ltd., China. The preparation materials of micro-capsule form mainly included stearic acid and glycerate. The preparation materials of micro-sphere form mainly included carboxymethyl cellulose and ethyl cellulose. MSB, menadione sodium bisulfite; MNB, menadione nicotinamide bisulfite.

**Table S2.** Effects of sources and forms of vitamin K_3_, and temperature/relative humidity on the retention of vitamin K_3_ in vitamin premixes.

| **T-H** | **Source** | **Form** | **Months (Time)** | | | |
| --- | --- | --- | --- | --- | --- | --- |
|  |  |  | **1** | **2** | **3** | **6** |
| 25℃/60% | MSB | crystal | 91^Ab^ | 83^Bb^ | 69^Cc^ | 44^Df^ |
|  |  | micro-capsule | 98^Aa^ | 88^Bab^ | 77^Cab^ | 56^Dcd^ |
|  |  | micro-sphere | 96^Aa^ | 87^Bab^ | 77^Cab^ | 59^Dcd^ |
|  | MNB | crystal | 95^Aab^ | 85^Bb^ | 75^Cabc^ | 54^Dcd^ |
|  |  | micro-capsule | 97^Aa^ | 89^Bab^ | 82^Ba^ | 60^Cbc^ |
|  |  | micro-sphere | 98^Aa^ | 94^Aa^ | 80^Ba^ | 67^Cab^ |
| 40℃/75% | MSB | crystal | 64^Ae^ | 37^Be^ | 22^Cf^ | 5^Dh^ |
|  |  | micro-capsule | 71^Ad^ | 50^Bd^ | 40^Ce^ | 11^Dgh^ |
|  |  | micro-sphere | 73^Ad^ | 48^Bd^ | 39^Ce^ | 15^Dg^ |
|  | MNB | crystal | 72^Ad^ | 72^Ac^ | 69^Ac^ | 38^Bb^ |
|  |  | micro-capsule | 74^Ad^ | 65^Bc^ | 58^Bd^ | 45^Cef^ |
|  |  | micro-sphere | 80^Ac^ | 73^Bc^ | 71^Bbc^ | 52^Cde^ |

^A, B, C, D^ Means in a row, with different superscripts, are different (*P* < 0.05). ^a, b, c, d, e, f, g, h^ Means in a column, with different superscripts, are different (*P* < 0.05). MSB, menadione sodium bisulfite; MNB, menadione nicotinamide bisulfite. T-H, temperature and relative humidity.

**Table S3.** Effects of sources and forms of vitamin K_3_ and choline on the retention of vitamin K_3_ in vitamin trace mineral (VTM) premixes.

| **Source** | **Form** | **Choline** | **Months (Time)** | | | |
| --- | --- | --- | --- | --- | --- | --- |
|  |  |  | **1** | **2** | **3** | **6** |
| MSB | crystal | - | 93^Aabc^ | 87^Babc^ | 84^Bc^ | 46^Cbc^ |
|  |  | + | 87^Ac^ | 83^Bbc^ | 75^Cd^ | 38^Dc^ |
|  | micro-capsule | - | 99^Aa^ | 88^Babc^ | 83^Cc^ | 50^Db^ |
|  |  | + | 98^Aa^ | 88^Bab^ | 84^Bc^ | 50^Cb^ |
|  | micro-sphere | - | 97^Aab^ | 88^Babc^ | 84^Bc^ | 62^Ca^ |
|  |  | + | 99^Aa^ | 94^Ba^ | 90^Ca^ | 50^Db^ |
| MNB | crystal | - | 98.^Aa^ | 80^Bc^ | 71^Ce^ | 38^Dc^ |
|  |  | + | 93^Aabc^ | 83^Bbc^ | 73^Cde^ | 37^Dc^ |
|  | micro-capsule | - | 94^Aabc^ | 92^Ba^ | 85^Cbc^ | 53^Dab^ |
|  |  | + | 95^Aab^ | 90^Bab^ | 85^Cbc^ | 50^Db^ |
|  | micro-sphere | - | 89^Abc^ | 87^ABabc^ | 84^Bc^ | 53^Cab^ |
|  |  | + | 96^Aab^ | 89^Bab^ | 88^Bbc^ | 52^Cab^ |

^A, B, C, D^ Means in a row, with different superscripts, are different (*P* < 0.05). ^a, b, c, d, e^ Means in a column, with different superscripts, are different (*P* < 0.05). MSB, menadione sodium bisulfite; MNB, menadione nicotinamide bisulfite.

**Figure S1.** The record of temperature and relative humidity during the storage of the vitamin trace mineral premixes.
